# In vivo competition and horizontal gene transfer among distinct *Staphylococcus aureus* lineages as major drivers for adaptational changes during long-term persistence in humans

**DOI:** 10.1186/s12866-018-1308-3

**Published:** 2018-10-22

**Authors:** Lars Langhanki, Petya Berger, Janina Treffon, Francesco Catania, Barbara C. Kahl, Alexander Mellmann

**Affiliations:** 1 Institute of Hygiene, University Hospital Münster, Robert-Koch Straße 41, Münster, 48149 Germany; 2 Institute of Med. Microbiology, University Hospital Muenster, Domagkstraße 10, 48149 Münster, Germany; 3 Institute for Evolution and Biodiversity Muenster, Hüfferstraße 1, 48149 Münster, Germany

**Keywords:** *Staphylococcus aureus*, Cystic fibrosis, Adaptation, Horizontal gene transfer, Genome sequencing

## Abstract

**Background:**

The airways of the majority of adolescent cystic fibrosis (CF) patients are persistently colonized or infected by *Staphylococcus aureus*. Using whole genome sequencing, we studied the evolutionary traits within a *S. aureus* population in the airways of a CF patient hypothesizing that horizontal gene transfer (HGT) and inter-bacterial interaction play a major role in adaptation during long-term persistence.

**Results:**

Whole genome sequencing of 21 *S. aureus* isolates spanning 13 years resulted in seven lineages defined by the *spa* types t012, t021, t331, t338, t364, t056, and t2351. Of these, the successfully persisting lineages t012 and t021 were closely related suggesting the evolution of t021 from t012, which was further corroborated by a nearly identical, syntenic set of mobile genetic elements. During transformation from t012 to t021, an increase of genomic changes including HGT from other *S. aureus* lineages was detected.

**Conclusions:**

In summary, our in vivo data enabled us to conceptualize an evolutionary model showing the impact of HGT and inter-bacterial interaction on bacterial long-term adaptation to the human host during CF.

**Electronic supplementary material:**

The online version of this article (10.1186/s12866-018-1308-3) contains supplementary material, which is available to authorized users.

## Background

About 20% of all humans are carriers of *Staphylococcus aureus*, which primarily colonizes the human skin and the mucosa of the anterior nares [1]. As a facultative pathogen, however, *S. aureus* can cause superficial skin infections as well as deeper infections like osteomyelitis, pneumonia and sepsis in addition to being one of the most important causes of nosocomial infections [2].

For colonization and long-term persistence in the human host, *S. aureus* has to adapt. With increasing duration of persistence, the bacterial population is exposed more and more to changing selective pressures exerted by the host immune system, to frequent therapeutic interventions, and to interference with other microorganisms [3, 4].

As a model system for the in vivo evolution and adaptation mechanisms of *S. aureus* during long-term persistence in the human host, we studied chronic airway infection in a cystic fibrosis (CF) patient. Patients suffering from this disease, which is caused by deleterious mutations affecting the CF transmembrane conductance regulator (CFTR) [5, 6], show, among other symptoms, an impaired mucociliary clearance in the airways brought about by the thickening of the mucus fluids on epithelial cells [7]. It has been proposed by Freedman et al. that dysfunction of the acinar tissue in CF may be due to an imbalance in the utilization of free fatty acids in the phospholipids of CF patients [8]. These changes cause recurrent and chronic bacterial infections, with *S. aureus* being especially one of the first pathogens colonizing and infecting the airways of CF-patients already in early childhood, followed by *Pseudomonas aeruginosa* and other Gram-negative non-fermenting bacteria [9–11]. However, the role that *S. aureus* and other organisms isolated from oropharyngeal cultures play in the progression of CF patients to respiratory failure has not been determined. Although the quality of life and life expectancy have increased during the last decades, more than 95% of their deaths are still related to respiratory insufficiency [12]. In spite of repeated antistaphylococcal therapy, *S. aureus* can persist in the airways for years, frequently developing phenotypic variants such as small-colony variants (SCVs) [13–16]. CF airways are an especially suitable setting for an in vivo study on evolution and adaptation, because within the airways, and especially in the mucus, bacteria are under enormous selective pressures. There, *S. aureus* has to cope with the presence of polymorphonuclear neutrophils [9], different ecological conditions in various regions of the lung [17–19], frequent antibiotic treatments, and competition with other bacteria [20]. Despite these pressures, the same clone of *S. aureus* is often able to persist in CF patients for months or even years [13]. The fact that *S. aureus* can persist for a long time emphasizes the ability of *S. aureus* to adapt to and to survive in the hostile CF airways environment. There are several known mechanisms underlying adaptive processes of *S. aureus* to CF airways: changes in the protein A gene (*spa*) repeat region [21], phage insertions and excisions [22] as well as the emergence of SCVs [15, 23, 24], which are especially thymidine-dependent in association with CF [23]. In addition, a significantly higher number of single nucleotide polymorphisms (SNPs) was detected in CF isolates compared to non-CF isolates within a study of different *S. aureus* lineages regarding their affiliation to mutator phenotypes [23].

It has been previously shown that HGT plays a key role in influencing the colonization of different *S. aureus* lineages across diverse habitats, including the ability for *S. aureus* to share genetic material with other bacterial pathogens (e.g. vancomycin-resistant enterococci) [25–27]. Especially mobile elements, which contain virulence and persistence genes, are exchanged by the bacteria [28]. For example, efficient transduction of penicillinase and tetracycline resistance plasmids by bacteriophages φ80α and φJB between clinical isolates belonging to the USA300 clone was reported [29]. In this way, mobile elements can contribute to *S. aureus* host adaptation [25]. Additionally, the role of HGT in *S. aureus* ruminant host adaptation was shown by Guinane and colleagues [26]. HGT also occurred extensively between human- and pig-associated *S. aureus* strains co-colonized on gnotobiotic piglets as described in the first in vivo HGT study in animals [27].

The findings described above prompted us to investigate a diverse *S. aureus* population originating from the airways of a single CF patient at different time points within a time span of 13 years [16, 30]. We hypothesize that a predominant clonal lineage, characterized by its *spa* type, underwent significant changes during more than a decade of persistence in the CF habitat. Furthermore, this lineage adapted to the host via HGT as the major mechanism driven by inter-bacterial competition. Using whole genome sequencing of instructive isolates (*n* = 21) representing seven clonal lineages, we were able to conceptualize an evolutionary scenario within the CF airways that could be a blueprint for adaptation processes during long-term persistence of bacteria in the human host.

## Methods

### *S. aureus* isolates

A total of 75 clinical *S. aureus* isolates were taken from the airways of a single CF patient between 1995 and 2008. None of the isolates exhibited a small colony variant (SCV) phenotype. These isolates were all taken during routine diagnostic efforts. All isolates were clustered together based on their *spa* types and assigned into lineages (predominant or rare) according to their *spa* types. *Spa* typing was first described as a highly discriminatory typing method based on the repeat pattern of the polymorphic region of *spa* [31, 32]; later a phylogenetic signal was attributed to the *spa* gene, which is highly concordant to multilocus sequence typing (MLST) and provides a higher level of discrimination than MLST by grouping strains of the same MLST sequence type (ST) into different spa types [33, 34]. We applied the following rules to choose instructive isolates for whole genome sequencing: (1) of the predominant lineages, the first isolate and- in case of repeated detections- only the first isolate per year and isolation site (deep throat swab, nasal swab or sputum) was sequenced. If isolates from the same lineage were obtained from different sites within the same year, they were compared and, in case of identical genotypes, only the first isolate was further investigated; (2) for all other lineages, only the first isolate was sequenced to investigate the extent of HGT between these lineages and the predominant lineages. A timeline of the sequenced isolates and their persistence are shown in Additional file 1; further details of the isolates are listed in Table 1.

**Table 1:** Characteristics of the analyzed *S. aureus* isolates

Isolate(source^a^)	Month/year of isolation	*spa* type (repeat pattern)	MLST
t012#95/“early” (T)	08/1995	t012 (15–12–16-02-16-02-25-17-24-24)	ST30
t012#97 (T)	12/1997	t012 (15–12–16-02-16-02-25-17-24-24)	ST30
t012#00 (T)	04/2000	t012 (15–12–16-02-16-02-25-17-24-24)	ST30
t021_2#02 (N)	01/2002	t021 (15–12–16-02-16-02-25-17-24)	ST30
t021#02 (T)	10/2002	t021 (15–12–16-02-16-02-25-17-24)	ST30
t021#04 (N)	07/2004	t021 (15–12–16-02-16-02-25-17-24)	ST30
t021_2#04 (T)	07/2004	t021 (15–12–16-02-16-02-25-17-24)	ST30
t021_2#06 (T)	02/2006	t021 (15–12–16-02-16-02-25-17-24)	ST30
t021_#06 (N)	05/2006	t021 (15–12–16-02-16-02-25-17-24)	ST30
t021#07 (T)	02/2007	t021 (15–12–16-02-16-02-25-17-24)	ST30
t021_2#07 (N)	02/2007	t021 (15–12–16-02-16-02-25-17-24)	ST30
t021#08 / "late" (S)	10/2008	t021 (15–12–16-02-16-02-25-17-24)	ST30
t338#95 (T)	05/1995	t338 (15–21–16-02-25-17-24)	ST30
t331_2#01 (T)	10/2001	t331 (08–16–34-13-17-34-16-34)	ST45
t331#01 (N)	10/2001	t331 (08–16–34-13-17-34-16-34)	ST45
t331#03 (N)	07/2003	t331 (08–16–34-13-17-34-16-34)	ST45
t331#06 (T)	05/2006	t331 (08–16–34-13-17-34-16-34)	ST45
t331#08 (T)	05/2008	t331 (08–16–34-13-17-34-16-34)	ST45
t364#03 (T)	07/2003	t364 (04–34–17-32-17-23-24)	ST3803
t2351III#04 (N)	07/2004	t2351(04–44–44-33-31-12-16-34-16-34-16-12-25-22-22-34)	ST34
t056#06 (T)	08/2006	t056 (04–20–12-17-20-17-12-17-17)	ST101

Isolation month/year and molecular typing results (*spa* typing and multilocus sequence typing [MLST]) of the analyzed *S. aureus* isolates. The first (“early”) and the last (“late”) isolate were previously subjected to complete genome sequencing [30]

^a^*N*, nasal swab, *T* deep throat swab, *S* sputum

### Whole genome sequencing

Isolates t012#95 (“early” isolate, sampled in 1995) and t021#08 (“late” isolate, sampled in 2008) were already fully sequenced to closed genomes in a previous study [30]. All other isolates (*n* = 19) were whole genome sequenced using the Nextera XT chemistry (Illumina Inc., San Diego CA, USA) for either a 100 bp paired-end sequencing run on an Illumina HiScan SQ sequencer (isolates t012#97, t012#00, t021#04, t021#06) or a 250 bp paired-end sequencing run on an Illumina MiSeq sequencer (all other isolates) in accordance to the manufacturer’s recommendations (Illumina). The “#” symbol in the strain IDs refers to the year of collection of the respective isolate. After quality trimming, the resulting reads were de novo assembled using the CLC Genomics Workbench 8.0.5 (CLC Bio, Qiagen, Venlo, The Netherlands) with default parameters and the consensus sequences were annotated using RAST NMPDR 2.0 (Rapid annotation using Subsystem Technology) [35]. Raw reads and annotated contig sequences are deposited at European Nucleotide Archive (ENA) under study accession number PRJEB22600.

### Bioinformatic analyses for between-strain comparisons

Based on *spa* types, the relatedness of the isolates was investigated with the Based Upon Repeat Pattern (BURP) algorithm [36] using default parameters “Exclude *spa* types that are shorter than 5 repeats ”and “*spa* types are clustered if cost is less or equal 4”. Related *spa* types were grouped into *spa* clonal complexes (*spa*-CC), where *spa* types are grouped together in a pairwise comparison depending on the minimal number of steps of evolution, i. e. “costs”, that are necessary to change the repeat region from one to another *spa* type.

For an unbiased comparison of isolates on whole genome level, the de novo assembled contigs or - in case of the t012 and t021 isolates - complete genome sequences were used as input sequences for generation of a k-mer based unrooted UPGMA tree with the aid of the CLC Genomics Workbench. The k-mer size was set to 15 and the distance calculation method was “Mahalanobis”.

### Mapping and extraction of whole genome consensus sequences of the t012/t021 lineage

The de novo assembled contigs of the twelve t012 and t021 isolates t012#97, t012#00, t021#02, t021_2#02, t021#04, t021_2#04, t021#06, t021_2#06 t021#07 and t021_2#07 (Table 1) were initially aligned to the complete genome sequence of the early or the late isolate (t012#95 and t021#08, respectively) using Mauve [37] (version Snapshot_2015-02-13). These alignments were subsequently reordered with the function “order contigs” to evaluate if the respective isolate was more closely related to either the early or the late isolate. Finally, the CLC Genomics Workbench was used to map the reads of the t012/t012 isolates to the sequentially closest reference isolate. By this approach, nearly fully closed consensus sequences could be achieved after the mapping. These sequences were then annotated again with RAST [35]. The small number of unmapped reads were de novo assemblied to exclude the possibility to miss novel sequences of the isolates that were neither present in the early nor in the late isolate.

### Detection of genomic differences and d_N_/d_S_ calculation

Detection of SNPs and other mutation events such as insertions and deletions (InDels) was performed with the program Breseq 0.27.1, which is a computational pipeline for finding mutations relative to a reference sequence in short-read DNA re-sequencing data for haploid microbial-sized genomes [38]. Depending on the closer relationship to either the early (t012#95) or the late (t021#08) isolate, we used the respective genome sequence as the reference sequence for a pairwise comparison with each of the remaining t012 and 021 isolates. From the output, all larger mutation events exceeding a length of 20 bp were additionally controlled by manual inspection of the Mauve alignment of the respective isolates and by a pairwise alignment of the respective contigs with the reference sequence of either the early (t012#95) or the late (t021#08) isolate using CLC Genomics Workbench. To further verify the results of Breseq, detection of mutations was also done via the “Basic Variant Detection” implemented in the CLC Genomics Workbench. For all non-t012/021 isolates, detection of mutations was only performed by using the Basic Variant Detection within the CLC bio Genomics Workbench, because for these isolates finished genome sequences were not available for comparison. Moreover, all genomic differences among all isolates of the t012/t021 lineages were investigated in detail in a pairwise comparison using Mauve. Finally, to investigate the origin of newly inserted genomic regions, we searched for these sequences within all other lineages via the BLAST Align Sequences Nucleotide tool (https://blast.ncbi.nlm.nih.gov/Blast.cgi). Genes, which were not present within the other lineages, were queried against the NCBI nucleotide database (https://www.ncbi.nlm.nih.gov/nucleotide?cmd=search) to further elucidate their origin. Calculation of the d_N_/d_S_ values, which are commonly used to estimate the balance between neutral mutations, purifying selection, and beneficial mutations, was done with the Ka/Ks Calculator 2.0 [39] using the method of Nei and Gojobori [40].

## Results

### Characterization of genomic variability among the *S. aureus* population

Initially, the relatedness among all *S. aureus* isolates (*n* = 21) was investigated by comparing their *spa* types with the BURP algorithm. Here, *spa* types t012, t021 and t338 clustered together in *spa*-CC021. Isolates of t012 and t021 exhibited the closest relationship; they differed only by a loss of a 24 bp repeat duplication in t021 [30]. The remaining *spa* types were unrelated to any other detected *spa* type and therefore classified as singletons (data not shown).

**Fig. 1:**
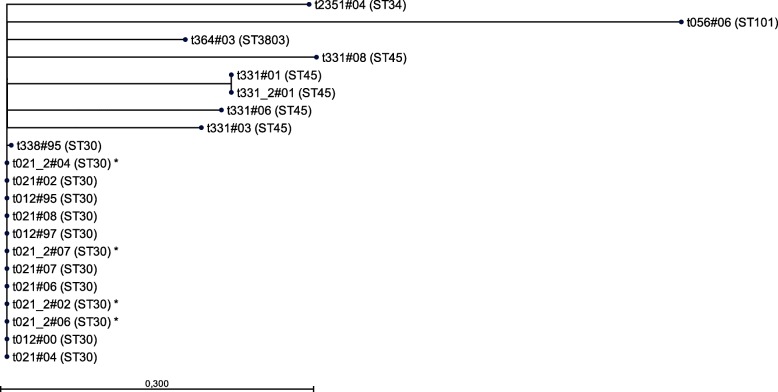
K-mer based unrooted UPGMA tree of all 21 *S. aureus* isolates used in this study. The tree was generated using CLC Genomics Workbench (k-mer size 15; distance calculation method was “Mahalanobis”) using the de novo assembled contigs or complete genome sequences of the respective isolates. The four isolates marked with an asterisk were isolated from another sampling source compared to the unmarked isolates from the same *spa* type obtained in the same year without exhibiting any differences on the genomic level (Additional File 5). Moreover, the MLST sequence types are given for each sample in parenthesis

Next, we compared all isolates on the whole genome level. By mapping the reads of all t012 and t021 isolates to the sequence of either the early or the late isolate, nearly fully closed consensus sequences could be obtained. As de novo assemblies of unmapped reads did not result in any genes or other meaningful sequences, they were excluded from further analysis. The possibility to map the reads against the finished genomes enabled us to characterize also mobile elements that frequently contain repeat or repetitive regions, which cannot be resolved using short read technology alone. The consensus sequences of the isolates t012#97 and t021#04 covered the complete sequences of the early and the late isolate, respectively. Only one and two nucleotides were missing in the isolates t021#07 and t021#06, whereas the consensus sequences of the isolates t012#00 and t021#02 were both missing one small genomic region, which consisted of 12 and 57 nucleotides, respectively. (hereinafter referred to as the t012/t021 lineage). Besides the BURP analysis, we determined the relatedness with a k-mer-based approach. Here, the unrooted UPGMA tree again showed the highest relatedness between the t012 and t021 *S. aureus* isolates taken from 1995 to 2008,whereas all other *spa* type lineages (t338, t364, t2351, t331 and t056) were not grouped into the same branch (Fig. 1). In consensus with the BURP clustering (results not shown), the t338 lineage exhibits the highest relatedness to the t012 and t021 lineage (hereinafter referred to as the t012/t021 lineage). Both independently showed the highest relatedness between the t012 and t021 isolates. Moreover, since the four pairs of t021 isolates recovered in the same year from different sampling sources, i. e. either nose or throat, were identical, isolates t021_2#02, t021_2#04, t021_2#06, and t021_2#07 were excluded from further analysis.

**Fig. 2:**
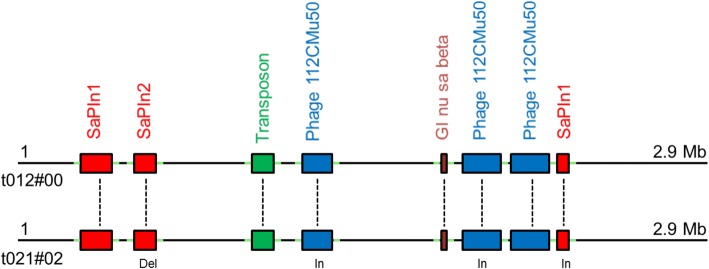
Comparison of mobile genomic elements of the isolates t012#00 and t021#02. The colored boxes on black horizontal lines (i. e. the chromosomes) represent the different mobile elements. Identical elements share the same color, are named accordingly, and are linked with a dashed line between the two isolates. Green bars flanking the mobile elements illustrate identical flanking genomic sequences in both isolates. Small insertions (In) and deletions (Del) in the pairwise comparison of the mobile elements are marked in the t021#02 isolate. Insertions within the shorter copy of SaPIn1 and the shortest copy of phage 112CMu50 are displayed in Fig. 3a and c, respectively. The deletions within SaPIn2 are shown in detail in Fig. 3b. The insertions within the longer copy of phage 112CMu50 are displayed in Fig. 3d

**Fig. 3:**
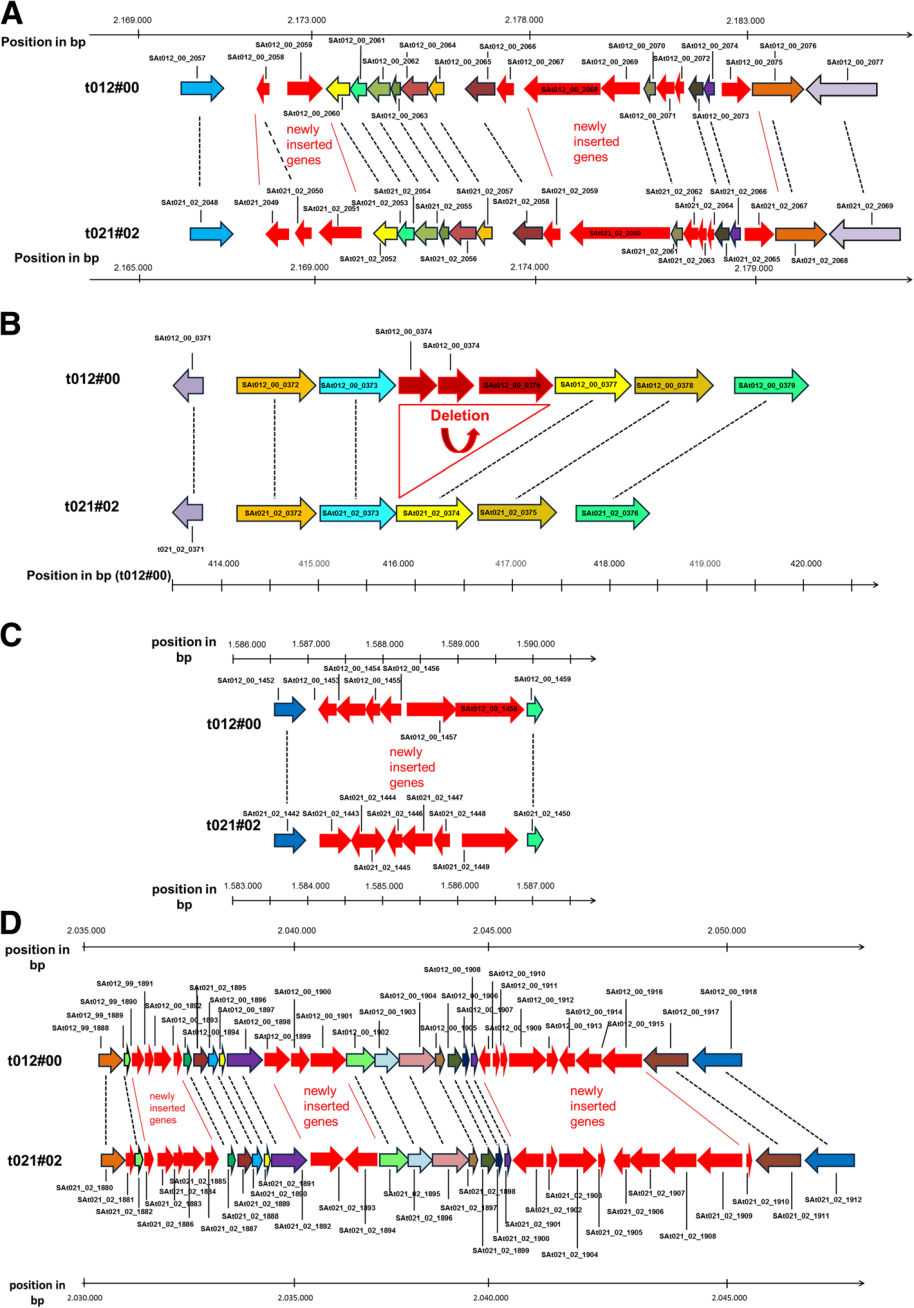
Schemes of the three structural differences on the genomic level between the isolates t012#00 and t021#02 within the mobile elements SaPIn1, SaPIn2, and SA bacteriophage 112C Mu 50 B. Genes on the forward strand are depicted as right-sided arrows; genes located on the reverse strand are depicted as left-sided arrows. Genes showing synteny between both genomes are highlighted with the same color and are linked with a dashed line. Genes that are present in just one of both genomes are colored in red. The locus tags of the genes and the genomic position in relation to the early (for t012#00) and late (for t021#02) genome sequence, respectively, are given and listed in Additional File 7. **a** Genomic differences within the *S. aureus* pathogenicity island SaPIn1. In both isolates, the islands are flanked by a gene encoding putative permease (upstream site) and by a gene encoding heat shock protein 60 family chaperone GroEL (downstream site). **b** Deletion of three genes encoding tandem lipoprotein within pathogenicity islandSaPIn2 in isolate t021#02. **c** Genomic differences within the shorter copy of phage SA bacteriophage 112C Mu 50 B, which is inserted in the identical genomic region. **d** Genomic differences within the longer copy of phage SA bacteriophage 112C Mu 50 B, which is inserted in the identical genomic region

**Fig. 4:**
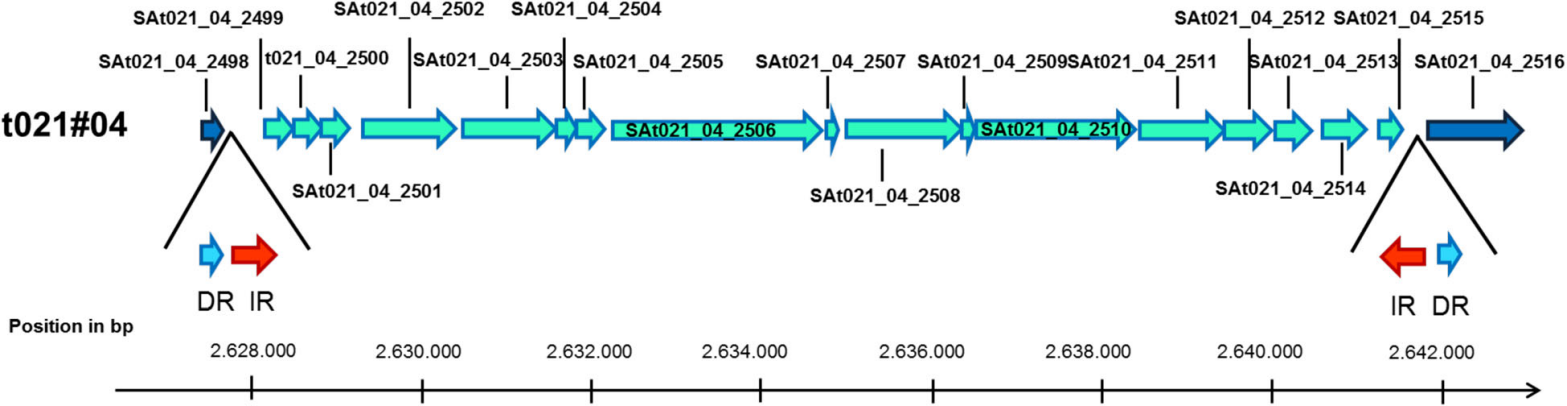
Genomic organization of the novel 14 kb transposon and its insertion site in isolate t021#04. Genes on the forward strand are depicted as right-sided arrows; genes located on the reverse strand are depicted as left-sided arrows. The transposon inserted into the gene encoding the carboxylesterase type B, whose parts border the insertion element both up- and downstream. The inserted genes are highlighted in turquoise; the inverted repeats (IR) are marked with red and the direct repeats (DR) are highlighted with light blue. For each gene, the locus tags are given (see also Additional File 6 for further details)

To elucidate whether the t021 isolates evolved from the t012 isolates, we performed an in-depth comparison at the whole-genome level. We detected differences in genome size (Additional file 2) partly due to the presence or absence of a plasmid. Specifically, the t012 isolates from 1995 and 2000 harbored - in contrast to the isolates t012#97 and t021#02-t021#08 - a 30 kb large plasmid. The remaining genome sequences showed a very high degree of similarity: All isolates of the t012 and t021 lineages contained the same set of mobile elements (“mobilome”), which included two non-identical copies of the pathogenicity island SaPIn1, one copy of SaPIn2, one genomic island called “nu Sa beta 2” and three non-identical copies of the phage “SA bacteriophage 112C Mu50B” (Fig. 2). Moreover, all isolates shared a 25.7 kb transposon in the region around 1.3 Mb (Additional file 3, Additional file 4). All mobile elements were inserted in the same genomic positions and were flanked by exactly the same genomic sequences in all isolates of both lineages. Among the two lineages, only minor differences were detected within the mobilome (Fig. 2). In particular, we detected small structural differences within the two Pathogenicity islands SaPIn1 (Fig. 3a) and SaPIn2 (Fig. 3b) as well as in two copies of the phage SA bacteriophage 112C Mu50B around the regions 1.583 Mb (Fig. 3c) and 2.035 Mb in the isolate t021#02 (Fig. 3d). Moreover, we found differences within another copy of the same bacteriophage in the region around 2.050 and 2.450 Mb (for details see Additional file 5). Only a few further genes were either deleted (*n* = 7) or inserted (*n* = 8) within other regions of the genome (Additional file 5). Overall, the t012 and t021 isolates share 2860 of the 2912 chromosomal ORFs (98.21%). In addition, all t021 isolates detected since 2004 shared an identical 14.2 kb insertion that was characterized as a DNA transposon containing 19 genes (Fig. 4 and Additional file 4). In all isolates, this fragment inserted into the gene encoding the carboxylesterase type B (SAt012_00_16_2499) within the region around 2.627 Mb of the t021#08 genome. The transposon is flanked by 28-bp inverted repeats, which are adjacent to 8-bp direct repeats. Interestingly, the identical 14.2-kb DNA transposon was also present within the five isolates of the t331 lineage (isolates t331#01, t331_2#01, t331#03, t331#06, and t331#08).

### An HGT-based model of the long-term persistence of *S. aureus* in CF airways

**Fig. 5:**
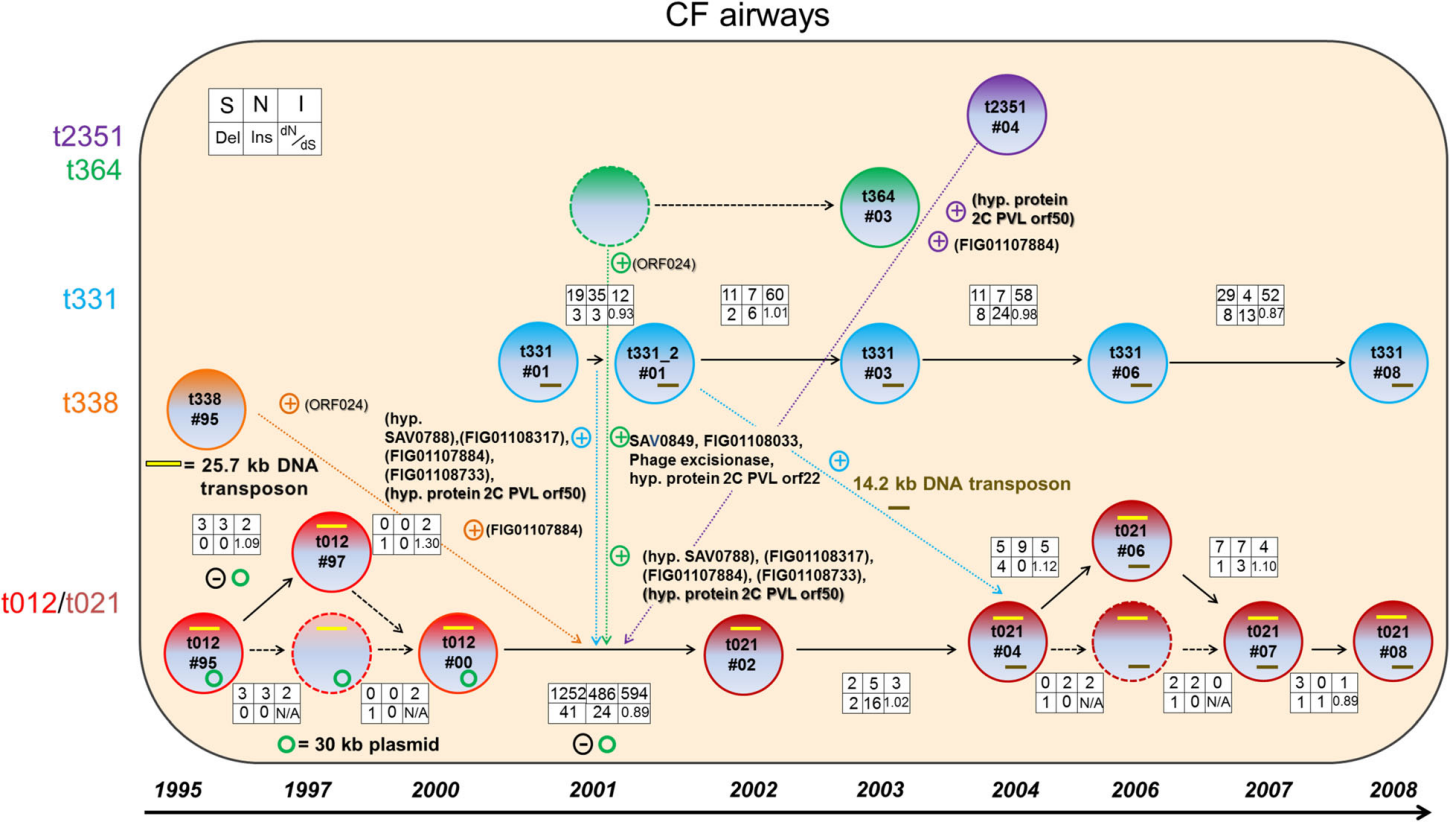
Model of the in vivo evolution and interactions of the CF *S. aureus* lineages from 1995 to 2008. The isolates within the airways are shown as colored circles; every lineage has its own color. Hypothetical isolates, which were not isolated but probably took place in the direct line of evolution, are depicted as dashed circles. Black arrows connect sequential isolates of the same lineage; dashed lines connect hypothetical isolates. Colored (**+**) and (**−**) indicate the acquisition or loss of genetic material. If genetic material was likely to be transferred from one lineage to another, dotted colored arrows show the assumed direction and the transferred material is listed. In case the origin was ambiguous, the transferred material is shown in brackets. In addition, the 25.7 kb DNA transposon (yellow line), the 30 kb plasmid (green circle), and the 14.2 kb DNA transposon (brown line) are shown. If applicable, boxes display in a pairwise comparison of consecutive isolates the number of differing synonymous (S), non-synonymous (N), or intergenic (I) SNPs, the number of insertions (In) and deletions (Del), and the d_N_/d_S_ value. N/A, not applicable

In order to put everything in an evolutionary context, we determined the number of SNPs and InDels between subsequent isolates to get a better understanding of how the evolution and adaptation of *S. aureus* could have arisen in this patient. The resulting evolutionary scenario of the *S. aureus* population in the airways of this CF patient, mainly relying on the assumption that newly acquired genes within the t012/t012 lineage were transferred via HGT, is shown in Fig. 5. The first *S. aureus* isolate with *spa* type t338 (t338#95) colonized the patient early in life (two years old) and was detected only once, while isolates of *spa* type t012, which were first detected 3 months later (t012#95), persisted the following 5 years with only minor changes apart from a temporary loss of the30 kb plasmid. Between 2000 and 2002, there was a clear increase of genomic alterations and SNPs between the isolates t012#00 and t021#02 as well as the change of the *spa* type from t012 into t021 and the loss of the 30 kb plasmid. During this interval a novel, different *S. aureus* lineage (t331) appeared. Five genes (SAV0788, FIG01108773, FIG01108317, FIG01107884, and hypothetical protein PVL orf50), which were newly acquired in the t012/t021 lineage at that time, were also present within the t331 lineage. These genes additionally appeared within the t364 lineage that was detected later, which made it impossible to determine from which lineage these genes were acquired by the t012/t021 lineage (Fig. 5). In contrast, four phage genes (FIG01108033, Phage excisionase, hypothetical protein PVL orf22, and SAV0788) were exclusively present within a hypothetical t364 isolate before they appeared in the t021 lineage. Some newly acquired genes, named ORF024 (t338), FIG01107884 (t338 and t2351), and hypothetical protein 2C PVL orf 50 (t2351) also appeared in the lineages given in brackets. Genes that were newly inserted into the t021 lineage but not present within any other lineage all stemmed from a staphylococcal background, which was shown by NCBI BLAST search results (Additional file 6).

In the years after 2002, more mutations appeared in the t021 lineage than in the first five years (1995–2000) of persistence (Fig. 5, Additional file 7). In general, these mutations were neither beneficial nor deleterious, as documented by the calculated d_N_/d_S_ ratios, which fluctuated around a value of one. Interestingly, the t331 lineage exhibited more mutations compared to the evolution of the t012/021 lineage in the years of co-existence starting in 2001 (Additional file 6 and Additional file 7). The lineages t012/t021 and t331 were the only ones that remained in the population after the initial interaction with various adaptation events. In contrast, t338 and t2351 were detected only once, t056 twice (08/2006 and 11/2007; only the first isolate was sequenced), and t364 three times (07/03, 10/2003 and 01/2004; similarly, only the first isolate was sequenced), respectively. A detailed list of all genomic alterations and SNPs are given in Additional files 4, 5, 6, 7 and 8.

## Discussion

The airways of CF with chronic bacterial infection represent a human in vivo setting for studying the long-term interaction and adaptation within the *S. aureus* populations. Here, we examined isolates that were recovered within a period of 13 years from the airways of an individual CF patient. These isolates consisted of seven different or closely related *S. aureus* lineages. Using in-depth genomic investigations, we were able to not only show genomic similarities among different lineages but could also conceptualize an evolutionary model wherein inter-bacterial co-existence and competition with subsequent HGT played a major role. Moreover, we detected the presence of dominant lineages that putatively exhibited a higher fitness demonstrated by their long-term persistence in comparison to other lineages that were present only for a limited time. As a sign of ongoing competition within the t012/t021 lineage, the number of SNPs increased during the introduction of novel lineages (t331 and t364 in 2001 and 2003, respectively), supporting the hypothesis that inter-bacterial interaction enhances mutations and HGT [20].

In this context, we initially asked whether the t021 isolates evolved from the t012 lineage. Alternatively, the t021 isolates may be a related but distinct newly emerging lineage that replaced the t012 lineage at some point in the years between 2000 and 2002. At first glance, the surge of genetic changes including (i) small structural genomic differences between these isolates within the mobilome (Fig. 2), (ii) insertions or deletions of single genes (Additional file 5), and (iii) the occurrence of a considerable number of SNPs between 2000 and 2002 (*n* = 2332; Fig. 5) supported the theory of a lineage replacement.

A number of facts however support the idea that the t021 isolates may have evolved from the t012 lineage. First, the t012 and t021 *spa* types differ only by the deletion of a single 24 bp repeat (in t021 compared to t012)**,** a mutation that can occur during persistence [21]. Furthermore, the high genomic similarity (Fig. 1) among the t012 and t021 isolates argues for the evolution of t021 from t012. Finally, and most importantly, both groups of isolates share a nearly identical mobilome located in exactly the same genomic positions (Fig. 2). This is the strongest argument in favor of the clonal heritage of t021 from t012, since an independent uptake of identical or at least highly similar mobile elements at the same genomic positions within two distinct lineages seems to be nearly impossible. In previous studies, an identical PFGE pattern was set as the criterion to declare the clonal heritage of different lineages [16]. Our study proposes a new, moleculargenetic definition of the clonal heritage: the lineages have to share the same mobilome with only minor InDels within the same genomic locations flanked by exactly the same sequences. This definition is further supported by the fact that mobile elements are also used as molecular markers to elucidate the common origin of species and evolutionary processes in eukaryotes [41, 42].

Assuming now that all t012 and t021 isolates were indeed the same clonal lineage, we investigated in detail all mutations that occurred in this lineage. Interestingly, between 2000 and 2002, we observed - besides the change of the *spa* type - an accumulation of genomic mutations between the isolates t012#00 and t021#02. Whereas SNPs might have arisen during host adaptation in the *S. aureus* genome, particularly in immune evasion pathways or specific host binding proteins [43], the accumulation of mutation events suggests that additional triggers for genomic changes were at least temporarily present. When we compared the affected genomic regions in the t012/t021 lineage with the genomic content of the other *S. aureus* lineages in our patient, we identified five genes, namely SAV0788, FIG01108773, FIG01108317, FIG01107884, and hypothetical protein PVL orf50, which could have been transferred from the t331 lineage into the t012/t021 lineage via HGT (Fig. 5). These five genes were also present in the t364 lineage in addition to the four further genes FIG01108033, Phage excisionase, hypothetical protein PVL orf22, and SAV0788 that were exclusively present in the t364 lineage; if these genes were descending from the t364 lineage, this scenario would expect that the t364 lineage was already present between 2000 and 2002 before its first detection in 2003. Furthermore, some of the genes named ORF024, FIG01107884, and hypothetical protein 2C PVL orf 50 could also stem from different lineages (t338, t364, and t2351) making the evolutionary scenario even more cross-linked (Fig. 5). In the case of the 14.2 kb DNA transposon, which was acquired by the t012/t021 lineage between 2002 and 2004, the t331 lineage is the only possible donor among our isolates. Although we could not completely trace back all “novel” genomic material within the t012/t021 lineage, which is one of the limitations of our study, we could verify via NCBI BLAST searches that the remaining genomic material originated from a staphylococcal background. Most likely, there was at least one undetected other *S. aureus* lineage present in the airways of this patient between 2000 and 2002 that served as a donor for genomic material. We can only speculate that the undetected lineage(s) were below the detection limit or not present at the sampling time points. Nevertheless, such lineage(s) could be the origin of additional genomic material as it was shown that HGT can occur in vivo even within the first four hours of co-colonization of two strains in an animal model [27].

After a quite stable phase during the first five years of *S. aureus* persistence, the situation changed with an increased number of mutations in the t012/t021 lineage and the presence of extensive HGT between 2001 and 2004. One explanation for this situation might be the presence of novel *S. aureus* lineages that started to compete with the existing ones thereby forcing them to further adapt. Interestingly, the t364 and t2351 lineages were only temporarily present, whereas the t331 lineage was regularly detected, albeit less frequently than the t012/t021 lineage, from 10/2001 until the end of the investigation in 2008. The increased number of SNPs further corroborated the interaction among the different lineages as elevated mutation rates due to inter-bacterial competition were already shown in different bacteria [20]. Interestingly, the t331 lineage also showed a high number of SNPs between the isolates indicating adaptation; however, there were no signs for HGT into the t331 lineage. From an evolutionary perspective, this scenario clearly evokes the assumption that the different lineages exhibited different levels of fitness or had yet unknown selective advantages in this setting. Thus, the t331 lineage could be also rated as a successful lineage as it was able to persist at least until the end of our investigation. For the t012/t021 lineage, which was the most successful lineage proven by its continuous persistence, the mutations seem to be at the very least beneficial for the adaptation to the hostile CF lung environment. In this context, the second limitation of our study, i. e. the lack of knowledge about the extent to which host and environmental factors such as the humoral and cellular host defense and antibiotics as well as possible undetected co-infections with other bacterial species also impact the in vivo evolution of the *S. aureus* population, should be mentioned. We only know for sure that - except frequent detections of *Haemophilus influenzae* - co-infecting species typical for CF patients, such as *P. aeruginosa*, *Stenotrophomonas maltophilia* or *Achromobacter xylosoxidans*, were not detected in this patient. An enhancing influence of these environmental factors on the mutation rate was suggested [44] as well as a selective advantage of mutators compared to non-mutators regarding the antibiotic treatment [20].

The finding that humans carry a diverse *S. aureus* population is not new [45], and the presence of a within-host evolution was already shown; however, in the study by Golubchik et al., the investigations were limited to isolates of the same clonal complex [45]. In contrast, we could demonstrate by comparative sequence analysis of all detected lineages within a population over more than a decade that genomic elements are indeed transferred between different lineages and remained in the donor underlining the benefit of these mutations that would have been otherwise purged in the case of any disadvantage. As described in a recent model, migration and HGT have a great impact to shape a microbial population [46]. All exchanged genes are located on mobile elements, which are known to contain virulence and persistence genes. It is one of the future challenges to discover the yet unknown function of these genes.

This model corroborates our findings, where the import of novel lineages (t331, t364, and t2351) prompted mutations on the one hand resulting in adaptation of successful lineages, i. e. t012/t021 and t331, and elimination of inferior lineages (i. e. t364 and t2351) on the other hand.

## Conclusions

In summary, we could show the importance of HGT in vivo leading to adapted *S. aureus* lineages. Although we could not determine the impact of host factors, this study is a blueprint for a better understanding of how and to what extend adaptation processes of *S. aureus* can occur during long-term persistence in the CF airways.

## Additional files

Additional file 1: Sampling scheme of the *S. aureus* isolates obtained within the time span between 1995 and 2008 from the airways of a single CF patient.Each star represents a sampling of *S. aureus* isolates with the respective *spa* type (y-axis). In the case of repeated sampling of isolates with the same *spa* type, the stars are connected with solid lines suggesting persistence. Only the isolates, which were included in further analyses, are shown. (PDF 36 kb)

Additional file 2: Genome sizes and of the *S. aureus* t012/t021 isolates. In addition, the presence (+) or absence (−) of the 30 kb plasmid is shown. (DOCX 24 kb)

Additional file 3: Genomic organization of the 25.7 kb transposon. Genes on the forward strand are depicted as right-sided arrows; genes located on the reverse strand are depicted as left-sided arrows. The transposon inserted into the gene encoding the carboxylesterase type B, whose parts border the insertion element both up- and downstream. The inserted genes are highlighted in turquoise; the inverted repeats (IR) are marked with red and the direct repeats (DR) are highlighted with light blue. For each gene, the locus tags are given (see also Additional file 6 for further details). (PDF 57 kb)

Additional file 4: Gene names, locus tags and position information characterizing the 14.2 kb DNA transposon and the 25.7 kb transposon, bordering genes are shown in light blue, the direct repeats are shown in light green, the indirect repeats in dark green. (XLSX 13 kb)

Additional file 5: Order and heritage of the genes differing between the isolates t012#00 and t021#02 (referring to Fig. 2). If the gene was present partially in another lineage it is marked with a % sign. Bordering genes are highlighted in light blue; genes, which are involved in the changes, are marked with green. (XLSX 24 kb)

Additional file 6: Blast results for all genes newly inserted into the t012/t021 lineage, which did not originate from one of the other lineages used in this study. The first respectively best blast match is shown for every gene. (XLSX 11 kb)

Additional file 7: SNPs and Indels between the consecutive t012 and t021 *S. aureus* isolates within the timespan between 1995 and 2008. The pairwise comparisons of the respective samples are listed in separate tabs and named accordingly. For each mutation, the nucleic acid changes and their position in relation to the genome sequence of t012#95 are given as well as the changes on amino acid level. Synonymous mutations are highlighted in green, non-synonymous mutations are marked in blue. A “*” displays a nonsense mutation (Stop). The changed nucleotide is highlighted in red. Deletions are labelled with a triangle; insertions are indicated by a plus. In addition, the orientation and if known, gene functions are given. Each tab contains the pairwise comparison of two consecutive isolates and is named accordingly. If there were no variants called, the table remained empty. The variants were detected with the program Breseq 0.27.1 (Deatherage and Barrick, 2014). NA, not applicable. (XLSX 131 kb)

Additional file 8: SNPs and InDels between the isolates of the t331 lineage. The mutations were detected with the “Basic Variant Detection” implemented in the CLC Genomics Workbench 8.0.5. SNP stands for single nucletiode polymorphism, MNP for multi nucleotide polymorphism; fs is the abbreviation of frameshift. For MNPs, every changed base is counted as a SNP in Fig. 5. It is also given if a SNP is synonymous or non-synonymous. If the SNP changes the amino acid, the new amino acid is reported. The nomenclature of the sequence variants can be viewed on http://www.hgvs.org/mutnomen/. (XLSX 30 kb)

## Abbreviations

BURP: Based Upon Repeat Pattern
CF: Cystic fibrosis
CFTR: CF transmembrane conductance regulator
HGT: Horizontal gene transfer
MNPs: Multi nucleotide polymorphisms
SCVs: Small-colony variants
SNPs: Single nucleotide polymorphisms
*spa*-CC: *Spa* clonal complexes
