# Supplementary figures and images for: In vivo competition and horizontal gene transfer among distinct Staphylococcus aureus lineages as major drivers for adaptational changes during long-term persistence in humans
Source: BMC Microbiol. 2018 Oct 22;18:152. doi: 10.1186/s12866-018-1308-3 (PMC6198438; doi:10.1186/s12866-018-1308-3)

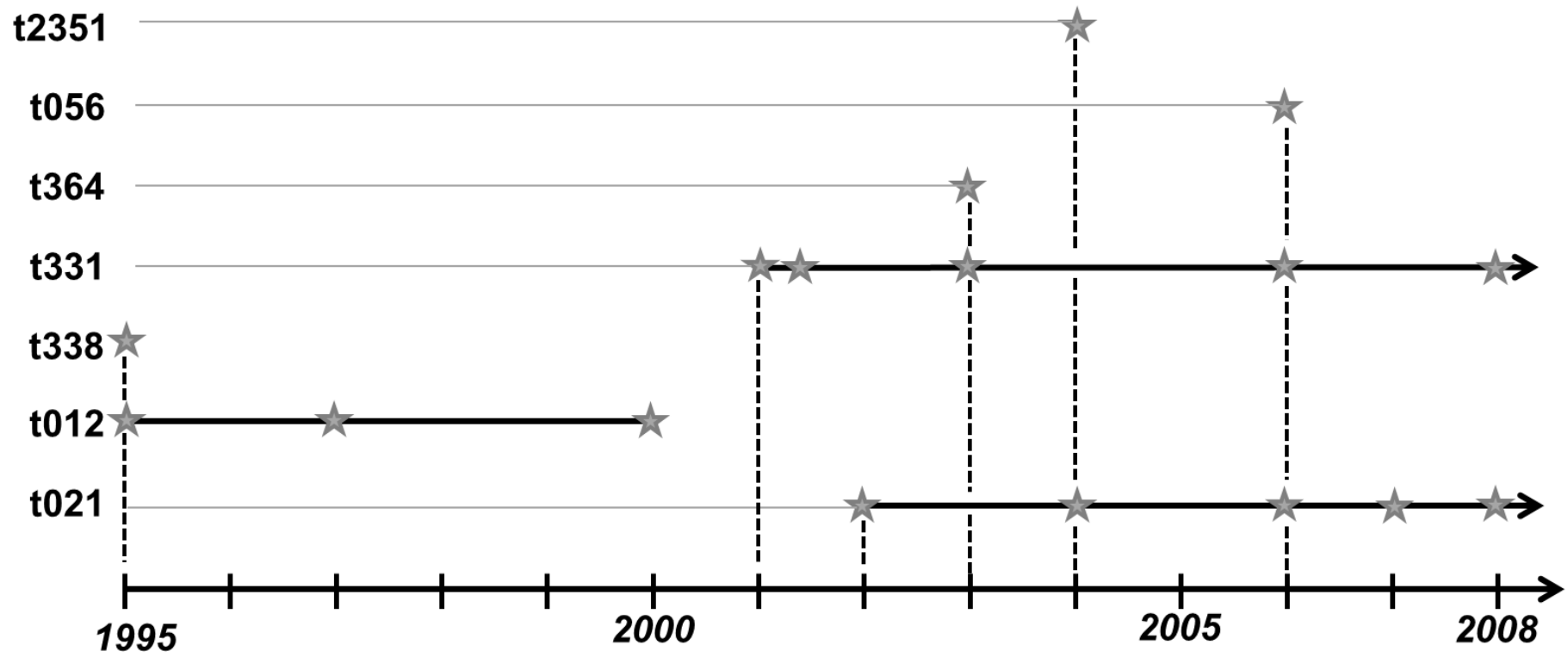

Supplement: Supplementary file 1 — Sampling scheme of the S. aureus isolates obtained within the time span between 1995 and 2008 from the airways of a single CF patient.Each star represents a sampling of S. aureus isolates with the respective spa type (y-axis). In the case of repeated sampling of isolates with the same spa type, the stars are connected with solid lines suggesting persistence. Only the isolates, which were included in further analyses, are shown. (PDF 36 kb) [file 12866_2018_1308_MOESM1_ESM.pdf]

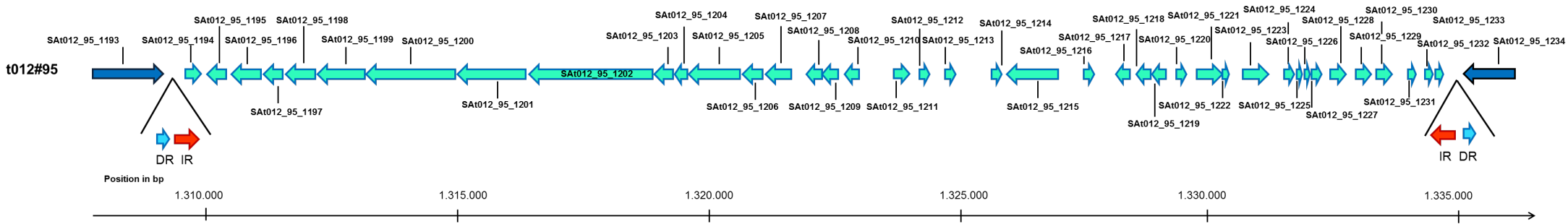

Supplement: Supplementary file 3 — Genomic organization of the 25.7 kb transposon. Genes on the forward strand are depicted as right-sided arrows; genes located on the reverse strand are depicted as left-sided arrows. The transposon inserted into the gene encoding the carboxylesterase type B, whose parts border the insertion element both up- and downstream. The inserted genes are highlighted in turquoise; the inverted repeats (IR) are marked with red and the direct repeats (DR) are highlighted with light blue. For each gene, the locus tags are given (see also Additional file 6 for further details). (PDF 57 kb) [file 12866_2018_1308_MOESM3_ESM.pdf]
