# Supplementary material for: In vivo competition and horizontal gene transfer among distinct Staphylococcus aureus lineages as major drivers for adaptational changes during long-term persistence in humans
Source: BMC Microbiol. 2018 Oct 22;18:152. doi: 10.1186/s12866-018-1308-3 (PMC6198438; doi:10.1186/s12866-018-1308-3)
Supplement: Supplementary file 2 — Genome sizes and of the S. aureus t012/t021 isolates. In addition, the presence (+) or absence (−) of the 30 kb plasmid is shown. (DOCX 24 kb) [file 12866_2018_1308_MOESM2_ESM.docx]

Additional File 2. Genome sizes and of the *S.aureus* t012/t021 isolates. In addition, the presence (+) or absence (-) of the 30 kb plasmid is shown.

| **Isolate** | **Genome size in bp** | **30 kb plasmid** |
| --- | --- | --- |
| t012#95 (“early“) | 2.937.161 | + |
| t012#97 | 2.907.074 | - |
| t012#00 | 2.937.149 | + |
| t021#02 | 2.908.025 | - |
| t021#04 | 2.922.214 | - |
| t021#06 | 2.922.209 | - |
| t021#07 | 2.922.214 | - |
| t021#08 (“late”) | 2.922.212 | - |
